# Physicians and nurses use and recommend dietary supplements: report of a survey

**DOI:** 10.1186/1475-2891-8-29

**Published:** 2009-07-01

**Authors:** Annette Dickinson, Nicolas Boyon, Andrew Shao

**Affiliations:** 1 Dickinson Consulting, LLC, 3432 Denmark Avenue, 350 St Paul, MN 55123, USA; 2 Ipsos Public Affairs, 1700 Broadway, New York, NY 10019, USA; 3 Council for Responsible Nutrition, 1828 L Street, N.W, Suite 510, Washington, D.C. 20036, USA

## Abstract

**Background:**

Numerous surveys show that dietary supplements are used by a large proportion of the general public, but there have been relatively few surveys on the prevalence of dietary supplement use among health professionals, including physicians and nurses. Even less information is available regarding the extent to which physicians and nurses recommend dietary supplements to their patients.

**Methods:**

An online survey was administered in October 2007 to 900 physicians and 277 nurses by Ipsos Public Affairs for the Council for Responsible Nutrition (CRN), a trade association representing the dietary supplement industry. The health professionals were asked whether they used dietary supplements and their reasons for doing so, and whether they recommend dietary supplements to their patients.

**Results:**

The "Life...supplemented" Healthcare Professionals Impact Study (HCP Impact Study) found that 72% of physicians and 89% of nurses in this sample used dietary supplements regularly, occasionally, or seasonally. Regular use of dietary supplements was reported by 51% of physicians and 59% of nurses. The most common reason given for using dietary supplements was for overall health and wellness (40% of physicians and 48% of nurses), but more than two-thirds cited more than one reason for using the products. When asked whether they "ever recommend dietary supplements" to their patients, 79% of physicians and 82% of nurses said they did.

**Conclusion:**

Physicians and nurses are as likely as members of the general public to use dietary supplements, as shown by comparing the results of this survey with data from national health and nutrition surveys. Also, most physicians and nurses recommend supplements to their patients, whether or not the clinicians use dietary supplements themselves.

## Background

Dietary supplements are used by the majority of U.S. adults, according to national surveys, including the National Health and Nutrition Examination Survey (NHANES) 1999–2000 [1]. Dietary surveys routinely show that population intakes of some vitamins and minerals are inadequate, and shortfall nutrients are specifically recognized in the *Dietary Guidelines for Americans *[2]. Supplemental intakes of some specific nutrients are recommended for some population groups not only in the *Dietary Guidelines *but also in some of the reports of the Institute of Medicine relating to Dietary Reference Intakes [3].

Some medical and nutrition experts have indicated that it would be prudent for adults to consume a daily multivitamin and perhaps additional amounts of some specific nutrients, in order to ensure adequate intakes and potentially help protect against some chronic diseases [4,5]. Increased intakes of calcium and vitamin D can help ensure bone health and reduce the risk of osteoporosis, and vitamin D is associated with protection against other conditions as well [6,7]. Supplemental intakes of other compounds such as the omega-3 fatty acids EPA and DHA have also been shown to have benefits for cardiovascular health and cognitive function and are currently being investigated in a large-scale government-sponsored clinical trial assessing their effect on the progression of eye disease [8]. Specialty supplements such as glucosamine and chondroitin sulfate have been found in many studies to improve joint health, and a variety of botanical products have functional and health benefits [9,10].

Health professionals including physicians and nurses are just as interested in healthy lifestyles as members of the general public and are just as likely to benefit from rational supplementation. There have been relatively few reported surveys of health professionals' use of dietary supplements, but the available surveys suggest that health professionals are as likely as other members of the public to use dietary supplements [11-17].

We report results of a survey regarding the use of dietary supplements by physicians and nurses, and the extent to which they recommend dietary supplements to their patients.

## Methods

The Council for Responsible Nutrition (CRN), a trade association representing the dietary supplement industry, contracted with Ipsos Public Affairs in 2007 to conduct the "Life...supplemented" Healthcare Professionals Impact Study (HCP Impact Study), intended as the first of a series of surveys of health professionals regarding their dietary supplement use and whether they recommend dietary supplements to patients. "Life...supplemented" is a consumer wellness initiative funded by a number of CRN member companies [18].

The survey questionnaire was administered online October 2–11, 2007 to 900 physicians and 277 nurses. The 900 physicians surveyed included 300 primary care physicians (PCP), 301 obstetricians/gynecologists (Ob/Gyn) and 299 other specialists (excluding pediatricians). The results for physicians were weighted to reflect the actual proportions of PCPs, Ob/Gyns and other specialists among all active physicians practicing in the United States as reported by the American Medical Association.

A total of 8,768 physicians and 3,028 nurses who are members of the All Global online panel were contacted and invited to take part in the survey. The All Global online panel is comprised of physicians, nurses and other healthcare professionals recruited by telephone to serve on a standing panel designed and used exclusively by All Global for market research studies [19]. For a fee paid to All Global, companies (mostly pharmaceutical companies and market research firms such as Ipsos) can obtain access to the panel in order to invite physicians and/or nurses who have "opted into" the panel to participate in a survey on a specific topic. The overall panel includes more than 200,000 subjects from the U.S. and Europe. Samples drawn from the panel can be designed to reflect the demographics of the U.S. population of physicians and nurses (or a subset of that population, e.g., specific specialists) as reported by the Medical Marketing Service for the American Medical Association and the National Sample Survey of Registered Nurses.

To protect against conflicts of interest, subjects included in our sample drawn from the All Global panel may not be affiliated with a market research company or advertising agency, a medical education company, or a pharmaceutical or dietary supplement company. Additional qualifications for participation in the current survey included: being a physician practicing any medical specialty except pediatrics; being a registered nurse or nurse practitioner; working primarily in an outpatient practice; and currently seeing more than 50 patients in their office each week.

A total of 1,378 physicians and 483 nurses accessed the survey. Nine hundred physicians and 277 nurses met all qualifying requirements and fully completed the survey. As an incentive to participate in the survey, healthcare professionals were offered a small honorarium. The honorarium amount (ranging from $25 to $50) and the response rates of 16% for both physicians and nurses are within the norms for online surveys among healthcare professionals.

In the survey instrument, dietary supplements were defined to include vitamins, minerals, herbals, botanicals or sports nutrition or specialty supplements. Respondents who said they used dietary supplements were asked if their use was regular, occasional, or seasonal. Occasional use was defined as taking supplements "throughout the year when I think of it or when the need arises." Seasonal use was defined as "taking them only during part of the year such as during the cold/flu season or allergy season."

## Results

Most of the physician respondents to the survey were male (83%), while most of the nurse respondents were female (94%). Of the physicians, 72% were in the age range of 40 to 59, as were 69% of the nurses.

The survey found that 72% of physicians and 89% of nurses used dietary supplements, when regular, occasional, and seasonal users are all included. Regular use of dietary supplements was reported by 51% of physicians and 59% of nurses; occasional use by 19% and 27% respectively; and seasonal use by 2% and 3% respectively. Fourteen percent of physicians and 8% of nurses said they had taken supplements in the past but no longer considered themselves supplement users; only 14% of physicians and 3% of nurses said they had never taken dietary supplements. For a summary of the main results, see Table 1.

**Table 1:** Use and recommendation of dietary supplements by physicians and nurses in the HCP Impact Study

	Physicians (n = 900)	Nurses (n = 277)
Percent that use dietary supplements (regularly, occasionally, or seasonally)	72%	89%

Percent *regular users *of dietary supplements	51%	59%

Recommend dietary supplements to patients	79%	82%

The dietary supplement product most commonly used was the multivitamin, with or without minerals. In our survey, 87% of physicians and 86% of nurses who used dietary supplements (regularly, occasionally, or seasonally) said they used a multivitamin, and many also used additional products. Among physicians, 51% said they were *regular *supplement users. This included 27% who said they regularly used a variety of dietary supplements and 24% who said they typically used only a multivitamin on a regular basis. Among nurses, 59% said they were regular supplement users. This included 32% who regularly used a variety of dietary supplements and 27% who typically used only a multivitamin on a regular basis.

Vitamins and minerals most commonly used, after multivitamins, included vitamin C, B complex, vitamin D, vitamin E, and calcium. The non-vitamin/mineral products most often used by physicians were green tea, fish oil, glucosamine, soy, flax seed, and chondroitin. The non-vitamin/mineral products most often used by nurses were green tea, fish oil, echinacea, glucosamine, and flax seed.

The most common reason why physicians and nurses in this survey said they took supplements was for overall health and wellness (40% of physicians and 48% of nurses), but more than two-thirds cited more than one motivation for using the products. For nurses, most of whom were female, bone health was the next most commonly mentioned motivation (46%). Many physicians as well as nurses also mentioned reasons relating to flu or colds (27% and 43%, respectively), heart health (24% and 22%), immune health (18% and 32%), joint health (22% and 30%), energy (20% and 29%), and musculoskeletal pain (14% and 21%).

In this survey, healthcare professionals were asked, "Do you ever recommend dietary supplements to patients?" A large proportion of physicians and nurses said they recommended supplements to their patients (79% of physicians and 82% of nurses). Among those who used supplements themselves, the proportion who recommended supplements to their patients was slightly higher (85% of physicians and 84% of nurses). However, even among those who did not use supplements themselves, there was still a high prevalence of recommending supplements to patients (62% of physicians and 67% of nurses).

The reasons for recommending dietary supplements to patients were similar to the physicians' and nurses' own reasons for using such products. The most common reason was for overall health and wellness (41% of physicians who recommend supplements and 62% of nurses who do). Physicians and nurses also recommended supplements for reasons related to bone health (41% and 58%, respectively), joint health (37% and 36%), flu or colds (24% and 39%), heart health (33% and 26%), immune health (19% and 36%), musculoskeletal pain (26% for both), and energy (19% and 25%).

Most physicians and nurses said they trusted professional journals (61% in each group) and clinical studies (56% of physicians and 57% of nurses) for reliable information about the dietary supplements they recommended to patients. Most physicians and nurses indicated they had not received any formal education or training on the subject of dietary supplements, but 75% of physicians and 79% of nurses indicated they would be interested in Continuing Medical Education regarding these products.

## Discussion

The level of usage of dietary supplements by physicians and nurses reported in this survey is similar to, or even higher than, the level of usage reported in some surveys of the general population. However, the reported level of usage in the general population is not consistent across surveys, in part because the exact nature of the question asked varies among surveys. Some surveys inquire about use within a short period of time such as the past two weeks or month, some ask about use within the past year, and some pose a general question about supplement use without specifying a time period. The first of these approaches will capture primarily regular users as well as some occasional users, while the last approach will capture virtually all supplement users. Some surveys inquire only about vitamin/mineral supplement use and some cover a broader range of dietary supplements.

The HCP Impact Study asked whether respondents took dietary supplements regularly, occasionally, seasonally, in the past, or never. The question was not limited to vitamin/mineral supplements, but included all types of dietary supplements. The prevalence of *regular *dietary supplement use among physicians and nurses (51% and 59%, respectively) was similar to the prevalence of use reported among adults in NHANES 1999–2000, where 52% of adults surveyed said they had taken supplements in the past month [1]. The NHANES question about usage within the last month captures mostly regular users. In the NHANES 1999–2000 study, the prevalence of supplement usage was higher in subgroups of adults more nearly comparable to the health professionals in the HCP Impact Study, in terms of age and education: usage was 56% among adults in the age range 40 to 60 and 62% among adults with more than a high school education. [Table 2] A survey of a large multiethnic cohort reported that 58% of men and 72% of women used any of eight dietary supplements regularly (at least once a week) [20].

**Table 2:** Percent dietary supplement users, HCP Impact Study compared to NHANES 1999–2000 and the CRN Consumer Confidence Survey 2007

	HCP Impact Study, Physicians (n = 900)	HCP Impact Study, Nurses (n = 277)	NHANES, 1999–2000, Adults (n = 4,862)	CRN Consumer Confidence Survey, 2007 (n = 2,153)
Percent using dietary supplements, overall (regular, occasional, seasonal)	72%	89%	--	68%

Percent regular users	51%	59%	52% overall; 56% among ages 40 to 60; 62% among those with 12+ years education	52%

A 2007 consumer survey conducted for the Council for Responsible Nutrition (CRN Consumer Confidence Survey) also provides information about current dietary supplement use in the general population [21]. In the CRN survey, 68% of the adult population identified themselves as dietary supplement users (regular, occasional, or seasonal), as compared to 72% of physicians and 89% of nurses in the HCP Impact Study. [Table 2]

Other surveys of dietary supplement use among health professionals have reported levels of usage generally similar to those observed in the HCP Impact Study. In most cases, the appropriate comparison is to our findings for *regular use *(51% for physicians and 59% for nurses). A recent survey of medical students found that 50% of male medical students and 63% of female medical students had used a vitamin or mineral dietary supplement in the month prior to the survey [11]. In a survey of women physicians, it was reported that 64% used vitamin or mineral supplements at least occasionally, and 47% of the women used a vitamin or mineral supplement at least 5 days a week [12]. Two surveys of health professionals enrolled in an online course on dietary supplements reported high levels of supplement use (over 80%), perhaps reflecting the interest that led them to enroll in the course [13,14]. A small convenience sample of pharmacists found that just over half (52.9%) reported taking dietary supplements [15]. Similar levels of dietary supplement use have been reported among directors of dietetic internship programs (53%) and dietetic interns (43%), and among Registered Dietitians in the state of Washington (nearly 60%) [16,17].

There are numerous published reports on the use of multivitamins and various specific nutrients from very large and longstanding cohorts of health professionals that have been the subject of many investigations of diet/disease relationships by researchers at Harvard University. The reports relating to multivitamin use are most relevant for comparison to our survey results. In our survey, 24% of physicians said they typically used only a multivitamin on a regular basis, and an additional 27% said they regularly used a variety of supplements. Among nurses, 27% said they typically used only a multivitamin on a regular basis, and an additional 32% said they used a variety of supplements regularly. We do not have specific data regarding the prevalence of multivitamin use as part of the "variety" of supplements, but we know from these data that the regular use of multivitamins is in the range of 24 to 51% for physicians and in the range of 27 to 59% for nurses. In the Nurses' Health Study and the Health Professionals Follow-up Study (mostly dentists), use of multivitamins was reported by about one-third of participants in studies published in 2003 and 2008 [22,23]. These levels of multivitamin use are comparable to that observed in the NHANES 1999–2000 survey, where 35% of adults in the general population said they had used multivitamin supplements in the past month.

The use of dietary supplements can be viewed as one of several elements of a healthy lifestyle, since it is known that users of dietary supplements also tend to adopt other healthy habits, including control of body weight, engaging in moderate or vigorous physical activity, and not smoking [1]. In some nutrition surveys, users of dietary supplements have been shown to have somewhat higher nutrient intakes from food alone, indicating that they pay more attention to their diets. However, the magnitude of the difference in dietary nutrient intake is small, and the intakes of many users as well as nonusers of dietary supplements fall short of recommended levels, for a number of vitamins and minerals [24,25].

Respondents to this survey were physicians and nurses who volunteered to serve on a national panel created for the purpose of participating in market research and later also volunteered to participate in this particular survey relating to dietary supplements. These respondents therefore are likely to be self-selected to include healthcare professionals with an interest in dietary supplements. The level of overall dietary supplement use in our survey is relatively high (72% in physicians and 79% in nurses), but the level of regular use (51% in physicians and 59% in nurses) is comparable to that found in other surveys.

It may appear surprising that physicians and nurses are as likely as the general population to be using dietary supplements, given the negative views sometimes expressed editorially in medical journals [26]. Much of the criticism of dietary supplements arises from concern about fringe products and exaggerated claims and does not necessarily relate to core products such as those most commonly used both by consumers and by healthcare professionals, namely multivitamins, calcium and other single nutrients, omega-3 fatty acids, glucosamine, and the more common botanicals. Indeed, articles affirmatively recommending multivitamins and some other dietary supplements appear in the same medical journals that are inclined to be editorially critical [4,5]. Physicians and nurses, as well as lay consumers, are exposed to these divergent views and must make their own decisions regarding their personal approach to wellness. The majority opt to use dietary supplements.

Most respondents in our survey said they had not received education or training about dietary supplements and expressed an interest in Continuing Medical Education on this topic. There has been a longstanding concern that medical education fails to provide practitioners with a sound basis for evaluating the role of nutrition in health and disease [27]. Providing more nutrition education in medical schools and increasing the availability of Continuing Medical Education relating to nutrition, including discussion of the role of dietary supplements, would be beneficial for physicians and nurses as well as for the patients they treat and serve.

## Conclusion

The HCP Impact Study shows that physicians and nurses are as likely as other members of the adult population to use dietary supplements, and further shows that most physicians and nurses recommend dietary supplements to their patients. However, most physicians and nurses in this survey indicated that they had not received any formal education or training on the subject of dietary supplements and expressed an interest in Continuing Education regarding these products. There is a need for expanded medical education regarding the general topic of nutrition as well as the more specific topic of dietary supplements.

## Abbreviations

CRN: Council for Responsible Nutrition; HCP Impact Study: "Life...supplemented" Healthcare Professionals Impact Study; NHANES: National Health and Nutrition Examination Survey.

## Competing interests

Dickinson is a consultant to the Council for Responsible Nutrition (CRN), and was formerly a VP and President of the association. Shao is a VP of CRN. Boyon is SVP with Ipsos Public Affairs, which conducted the survey for CRN.

## Authors' contributions

AD prepared the original draft of the article, for subsequent evaluation and elaboration by all of the authors working collaboratively. NB participated in the design and administration of the survey, including the data analysis. All of the authors provided meaningful insight regarding the results and implications of the survey findings, in the context of previously reported research, and all approved the final version of the article.

## References

[B1] Radimer K, Bindewald B, Hughes J, Ervin B, Swanson C, Picciano MF (2004). Dietary supplement use by U.S. adults: Data from the National Health and Nutrition Examination Survey, 1999–2000. Am J Epidem.

[B2] (2005). U.S. Department of Health and Human Services and U.S. Department of Agriculture. Dietary Guidelines for Americans.

[B3] Institute of Medicine (1998). Dietary Reference Intakes for thiamin, riboflavin, niacin, vitamin B-6, folate, vitamin B-12, pantothenic acid, biotin, and choline.

[B4] Willett WC, Stampfer MJ (2001). Clinical practice: What vitamins should I be taking, doctor?. N Engl J Med.

[B5] Fletcher RH, Fairfield KM (2002). Vitamins for chronic disease prevention in adults; clinical applications. JAMA.

[B6] Department of Health and Human Services (2004). Bone health and osteoporosis: A report of the Surgeon General.

[B7] Holick MF (2006). High prevalence of vitamin D inadequacy and implications for health. Mayo Clin Proc.

[B8] Gruppo Italiano per lo Studio della Sopravvivenza nell'Infarto miocardico (1999). Dietary supplementation with n-3 polyunsaturated fatty acids and vitamin E after myocardial infarction: results of the GISSI_Prevenzione trial. Lancet.

[B9] Clegg DO, Reda DJ, Harris CL, Klein MA, O'Dell JR, Hooper MM, Bradley JD, Bingham CO, Weisman MH, Jackson CG, Lane NE, Cush JJ, Moreland LW, Schumacher HR, Oddis CV, Wolfe F, Molitor JA, Yocum DE, Schnitzer TJ, Furst DE, Sawitzke AD, Shi H, Brandt KD, Moskowitz RW, Williams HJ (2006). Glucosamine, chondroitin sulfate, and the two in combination for painful knee osteoarthritis. New Engl J Med.

[B10] Blumenthal M, Senior Editor (2003). The ABC Clinical Guide to Herbs.

[B11] Spencer EH, Bendich A, Frank E (2006). Vitamin and mineral supplement use among U.S. medical students: A longitudinal study. J Am Diet Assoc.

[B12] Frank E, Bendich A, Denniston M (2000). Use of vitamin-mineral supplements by female physicians in the United States. Am J Clin Nutr.

[B13] Gardiner P, Woods C, Kemper KJ (2006). Dietary supplement use among health care professionals enrolled in an online curriculum on herbs and dietary supplements. BMC Complement Altern Med.

[B14] Kemper KJ, Gardiner P, Woods C (2007). Changes in use of herbs and dietary supplements (HDS) among clinicians enrolled in an online curriculum. BMC Complement Altern Med.

[B15] Howard N, Tsourounis C, Kapusnik-Uner J (2001). Dietary supplement survey of pharmacists: personal and professional practices. J Altern Complement Med.

[B16] Box S, Creswell B, Hagan DW (2001). Alternative health care education in dietetic training programs: A survey of perceived needs. J Am Diet Assoc.

[B17] Worthington-Roberts B, Breskin M (1984). Supplementation patterns of Washington state dietitians. J Am Diet Assoc.

[B18] Life...supplemented consumer wellness program. http://www.lifesupplemented.org/.

[B19] All Global online panel of physicians and other healthcare professionals. http://www.allglobal.com/Online/index.html.

[B20] Foote JA, Murphy SP, Wilkens LR, Hankin JH, Henderson BE, Kolonel LN (2003). Factors associated with dietary supplement use among healthy adults of five ethnicities: the Multiethnic Cohort Study. Am J Epidemiol.

[B21] Council for Responsible Nutrition (2007). More consumers consider themselves "regular" supplement users, annual survey results show. http://www.crnusa.org/CRN_PR_100407_ConsumerConfidence.html.

[B22] Rimm EB, Willett WC, Hu FB, Sampson L, Colditz GA, Manson JE, Hennekens C, Stampfer MJ (1998). Folate and vitamin B-6 from diet and supplements in relation to risk of coronary heart disease among women. J Am Med Assn.

[B23] Merchant AT, Hu FB, Spiegelman D, Willett WC, Rimm EB, Ascherio A (2003). The use of B vitamin supplements and peripheral arterial disease risk in men are inversely related. J Nutr.

[B24] Murphy SP, White KK, Park S-Y, Sharma S (2007). Multivitamin-multimineral supplements' effect on total nutrient intake. Am J Clin Nutr.

[B25] Sebastian RS, Cleveland LE, Goldman JD, Moshfegh AJ (2007). Older adults who use vitamin/mineral supplements differ from nonusers in nutrient intake adequacy and dietary attitudes. J Am Diet Assoc.

[B26] DeAngelis CD, Fontanarosa PB (2003). Drugs alias dietary supplements. JAMA.

[B27] Adams KM, Lindell KC, Kohlmeier M, Zeisel SH (2006). Status of nutrition education in medical schools. Am J Clin Nutr.

